# If It’s Not One Thing, It’s Another: An Inverse Relationship of Malignancy and Atherosclerotic Disease

**DOI:** 10.1371/journal.pone.0126855

**Published:** 2015-05-22

**Authors:** Matthew Li, Michael J Cima, Danny A. Milner

**Affiliations:** 1 Harvard-MIT Division of Health Science and Technology, Harvard Medical School, Massachusetts Institute of Technology, Cambridge, Massachusetts, United States of America; 2 Department of Materials Science and Engineering, Massachusetts Institute of Technology, Cambridge, Massachusetts, United States of America; 3 Department of Pathology, Brigham and Women’s Hospital, Boston, Massachusetts, United States of America; University of Palermo, ITALY

## Abstract

Atherosclerosis and malignancy are pervasive pathological conditions that account for the bulk of morbidity and mortality in developed countries. Our current understanding of the patholobiology of these fundamental disorders suggests that inflammatory processes may differentially affect them; thus, atherosclerosis can be largely driven by inflammation, where as cancer often flourishes as inflammatory responses are modulated. A corollary of this hypothesis is that cancer (or its treatment may significantly attenuate atherosclerotic disease by diminishing host inflammatory response, suggesting potential therapeutic approaches. To evaluate the relationship between cancer and cardiovascular atherosclerotic disease, we assessed 1,024 autopsy reports from Brigham and Women’s Hospital and performed correlative analyses on atherosclerotic severity and cancer prevalence. In gender- and age-matched populations, there is a statistically significant inverse correlation between history of malignancy and autopsy-proven atherosclerotic disease. In a second analysis, we evaluated 147,779 patients through analysis of the Harvard Catalyst SHRINE database and demonstrated a reduced non-coronary atherosclerotic disease rate: control (27.40%), leukemia/lymphoma (12.57%), lung (17.63%), colorectal (18.17%), breast (9.79%), uterus/cervix (11.47%), and prostate (18.40%). We herein report that, based on two separate medical records analysis, an inverse correlation between cancer and atherosclerosis. Furthermore, this correlation is not uniformly associated with anti-neoplastic treatment, suggesting that the inverse relationship may be in part attributable to an individual’s intrinsic inflammatory propensity, and/or to inflammation-modulatory properties of neoplasms.

## Introduction

Atherosclerosis is an inflammation driven disease characterized by arterial wall thickening associated with local hyperplastic growth and accompanied by immune cell infiltration and accumulation of lipids. Linked to events such as myocardial infarction and stroke, it is considered the causative factor for most cardiovascular diseases (CVD) and a leading candidate for worldwide mortality with roughly 13.5 million associated deaths in 2008 [1].

Several modes of interventional therapies are commonly practiced towards combating this highly pervasive disease: lifestyle changes (diet and exercise), pharmaceutical agents, and invasive vascular surgeries in the most severe cases [2]. These therapies are generally thought to be associated with improved cardiovascular risks, however, a complete understanding of their mechanisms of actions as well as inherent underlying pathological development is still lacking [3,4,5]. Recent years have demonstrated that aggressively lowering lipid levels does not have as profound an effect as once believed while the efficacious nature of statins now appears to stem from their anti-inflammatory properties; both these notions begin to challenge the long held belief that lipid control and attenuation would be the predominant path towards atherosclerotic improvement [5–11]. This has been further substantiated with the use of C-Reactive Protein (CRP), a marker of global inflammation, as a better diagnostic tool than lipid levels [12]. The World Health Organization (WHO) estimates an increase to nearly 24 million CVD associated deaths by 2030 [1]. New molecular pathways and mechanisms must be uncovered in order to recognize the critical underpinnings of atherosclerotic development and progression.

Malignant cancers are considered a diverse category of diseases and encompass etiologies ranging from genetic predispositions, exposure to environmental factors, and adverse responses to inflammation. Direct associations between cancer and causative factors, such as prolonged estrogen exposure in estrogen-receptor positive breast carcinomas, exist but, unfortunately, it is not possible to state a “unifying” theory for the pathological origin of cancer. Although cancer is a leading cause of morbidity and mortality, immune surveillance provides a crucial control mechanism in keeping malignant incidences at some minimum. Hematological malignancies, including lymphomas and leukemias, are comprised of aberrant cells that normally initiate and control the inflammatory response—this may explain a greater effect in these cancers on atherosclerosis as herein reported. Immunosuppression, whether congenital, acquired, or iatrogenic, also produces malignancies in these populations more frequently than in normal hosts.

We propose that the baseline human physiology can be either pro-, balanced, or anti-inflammatory; and/or that neoplastic populations also possess pan-inflammatory characteristics (more importantly, key anti-inflammatory traits). The patients may exhibit secreted factors or indirect immune modulatory processes that provide the systemic anti-inflammatory traits. As a logical extension, we suggest that atherosclerosis results, as data suggests, from pro-inflammatory individuals and, at the other end of the spectrum, anti-inflammatory individuals could have a higher predilection for cancers.

This hypothesized inverse relationship between atherosclerosis and malignancy builds off of anecdotal pathological observations as well as sparse reports in literature from more than half a century ago. Several reports dating back to the 1950s noted decreased atherosclerotic burden through autopsy analyses [13,14,15]. Though this phenomenon was documented, no substantial effort was made towards establishing a concrete mechanism of action. Further, the common presumption that “chemotherapy removes atherosclerosis” is dated, unsubstantiated, and not rational within the current understanding of atherosclerotic biology.

We evaluated data from Brigham and Women’s electronic medical records database and the Harvard Catalyst Shared Health Research Information Network (SHRINE) to assess the severity and prevalence of atherosclerotic disease as a function of cancer history and status.

## Methods

### Ethics statement

The institutional review board of the Brigham and Women’s Hospital reviewed and approved this study. All data were analyzed either in de-identified format or were from deceased patients (over a 10 year period) in whom written informed consent was not feasible.

### Autopsy dataset

The autopsy data set was chosen as a time point analysis for the history or current status of malignancy and definitively and completely defined atherosclerotic disease of all sites. This allowed for the assessment of two specific disease types regardless of the etiology of death. Final autopsy reports were obtained through the Brigham and Women’s Hospital Longitudinal Medical Records (BWH-LMR) database. Reports were manually analyzed from 2004–2012 for the following patient information: age, gender, history of cancer (yes/no), cancer type (if applicable), history of smoking (yes/no), hypertension (yes/no), type I/II diabetes mellitus (yes/no), aortic atherosclerosis (graded by severity), and coronary atherosclerosis (graded by severity).

The qualitative reports by pathologists of atherosclerosis status in autopsy cases were normalized and assigned a numerical severity grading. The grading scale consisted of 5 tiers: none, minimal, mild, moderate, and severe. Characteristics that were commonly assessed within reports consisted of percent vessel occlusion, severity of calcification, ulcerative status, and descriptive statements of “minimal”, “severe”, etc.

Exclusion criteria for reports included: only neurologic reports provided, no reported atherosclerosis status of aortic and coronaries, or patients having a history of more than one cancer.

### Imputation

Numerous reports indicated either aortic or coronary atherosclerosis in autopsy records. Although there can be discordance between aortic and coronary atherosclerotic disease, for purposes of statistical calculations, we made an assumption that atherosclerotic disease in the aorta and coronaries likely progress on a similar time and severity scale. Unreported atherosclerosis statuses of either the coronaries or the aorta were thus imputed to prevent loss of the data point. The missing information was assigned the same grading value as the one that was actually reported. The fallacy in such imputation is regression towards the mean of effect size evaluated. The validity of this imputation was confirmed by comparing the pre- and post-imputation effects on the model outputs (S1 and S2 Tables).

### Medical Records Cohort Dataset

Cohort information was obtained through the Harvard Catalyst Shared Health Research Information Network (SHRINE)—a web-based query tool capable of determining the aggregate number of patients at participating hospitals that meet a given set of inclusion/exclusion criteria. Our dataset was limited to a patient population over 50 years of age to avoid genetic mutations that lead to premature atherosclerotic and/or cancer syndromes. The dataset was further simplified to assess non-coronary associated atherosclerosis (cerebrovascular disease, aortic and peripheral arterial embolism or thrombosis, aortic, peripheral, and visceral artery aneurysms, and peripheral and visceral atherosclerosis). A total of 147,779 patients were available for analysis.

An out-group (patients with non-pathological fractures) was selected as a proxy for “population” prevalence of atherosclerosis; this was validated against a published rate for atherosclerosis. A broad test group panel of cancers was selected: male cancer (prostate), sex independent cancer (lung and colon), female cancers (breast, uterus/cervix), and leukemia/lymphomas. Analyses were performed through dual reciprocal searches. For a given category (i.e. lymphoma), we queried the complete dataset for those with lymphoma and non-coronary associated atherosclerosis and divided by those with lymphoma only, producing a percentage. The reciprocal search was performed on those with non-coronary associated atherosclerosis with lymphoma over the total.

### Statistical Analysis

All statistical analyses were performed through STATA Data Analysis and Statistical Software (StataCorp LP). Multivariate logistic regressions employed a jackknifing resampling technique in order to assess biases within the dataset. All regressions outputs are shown in S3 Table. The outcome variable was cancer being tested for association with aortic/coronary atherosclerosis. The covariates for the models were age, gender, history of smoking, hypertension, and diabetes. All models were initially regressed using the entirety of the aforementioned parameters. P-values for all variables and intermediate model states are shown in supporting information (S1, S2, and S3 Tables). Pseudo-r^2^ values for the model were computed. Model parameters were then further optimized on a per cancer subset basis as follows: the gender variable was not included in gender specific cancers; parameters were considered essential if removal resulted in a greater than 10% change in odds ratio or a greater than 5% increase in standard error.

#### Aortic and coronary atherosclerosis were assessed in separate models

Simple imputation was used to generate missing data, for aortic and coronary, to maximize the number of valid cases in the model. Imputation proceeded as follows: when data was available for aortic but not coronary, the aortic data was assigned to coronary; when coronary data was available but not aortic, coronary data was assigned to aortic; if both were missing, the case was excluded. The natural biological correlation of aortic and coronary atherosclerosis is variable, however, only 197 cases had values imputed in either direction (19%) and 58 cases were excluded (6%). The two outputs were highly correlated and, thus, only one was used per model as to not over fit the data.

Categorical analyses were performed on the following cancer groups: cancer, carcinoma, hematologic, sarcoma, neurologic, lung, breast, prostate, leukemia, lymphoma, and myeloma. Selection and subdivision into more specific cancers classes was dictated by sample number in which a minimum of n = 20 was set.

Within the SHRINE data, binomial proportion was used to assess the significance of differences in the cancer groups vs. the outgroup (fractures).

## Results

### Autopsy Results

Characteristics of the autopsy dataset are summarized in Table 1. A total of 1,024 final autopsy reports from Brigham and Women’s Hospital between 2004–2012 were analyzed, 502 (49.02%) were free of any cancer history while 522 (50.98%) had some history of cancer. There was a wide range of cancer subtypes, age, and prevalence of risk factors for atherosclerosis.

**Table 1:** Descriptive population statistics.

	Cancer		No Cancer	
	Female	Male	Female	Male
**# of Individuals**	210	312	218	284
**Age Range**				
<20	0	1	0	1
20–39	11	24	19	23
40–59	83	111	59	110
60–79	95	146	105	118
80+	21	30	35	32
**Smoking History**				
Unknown	121	180	127	171
No	55	65	41	39
Yes	34	67	50	74
**Hypertension**				
No	114	151	66	88
Yes	96	161	152	196
**Diabetes Mellitus**				
No	185	256	148	183
Yes	25	56	70	101

A total of 1,024 patient files were assessed over an eight-year time frame. Information on gender, age, history of smoking, hypertension, and diabetes were assessed along side atherosclerotic burden and cancer in our multivariate analysis.

The coronary artery disease score and the aortic atherosclerosis score showed modest agreement in the raw data set (42.39%) with Pearson correlation of 0.5864. After imputation, the agreement remained modest (53.94%) with identical correlation (0.5864). The correlation of the pre- and post-imputation scores for each category were 0.9997 (coronary artery disease) and 0.9996 (aortic atherosclerosis). In supporting information, we show the detailed OR for cancer and subtypes for both aortic and coronary atherosclerosis before and after imputation for which the ORs largely agree except as follows: Lung for aortic could not be calculated but was resulted after imputation and increased slightly for coronary; heme (grouped), neuro, and sarcoma cross 1.0 after imputation suggesting these results in a coronary model are not associated (but all held true in aortic) (S1 and S2 Tables).

Significant, negative correlations were noted between presence of cancer and atherosclerosis severity when controlled for standard population characteristics and atherosclerotic risk factors. The complete cancer group had an odds ratio of 0.468 (95% CI: 0.287–0.765) for aortic atherosclerosis and 0.772 (95% CI: 0.518–1.151) for coronary atherosclerosis. Comparatively, hematologic cancers (OR 0.478, 95% CI: 0.261–0.874), carcinoma (OR 0.566, 95% CI: 0.293–1.070), sarcoma (OR 0.561, 95% 0.112–2.799), and neurologic cancers (OR 0.301, 95% CI: 0.112–2.799) had similar effects on aortic atherosclerosis, Table 2. There appeared greater disparity in coronary atherosclerosis: hematologic (OR 1.047, 95% CI: 0.604–1.814), carcinoma (OR 0.596, 95% CI: 0.370–0.960), sarcoma (OR 0.869, 95% CI: 0.230–3.286), and neurologic (OR 1.182, 95% CI: 0.361–3.873), Table 3. Further sub-categorical analyses can be found in both Tables 2 and 3. Pseudo-r^2^ (ρ^2^) values for each model are shown in supporting information tables but ranged from 0.12 to 0.32 depending on the model (S1, S2, and S3 Tables). It should be mentioned that pseudo-r^2^ interpretations are quite different from standard r^2^ analyses. Values from 0.2–0.4 are said to have excellent fit characteristics where the ~0.15 range is fair [16].

**Table 2:** Aortic atherosclerosis, statistical results.

	Odds Ratio	Std Error	P	CI	ρ^2^	n
**Cancer**	0.468	0.117	0.002	0.287–0.765	0.2309	522
**Carcinoma**	0.566	0.184	0.080	0.293–1.070	0.2310	259
Breast	0.329	0.218	0.095	0.084–1.218	0.2840	23
Prostate*	.	.	.	.	0.2683	22
Lung	1.801	1.90	0.577	0.228–14.213	0.2585	50
**Heme**	0.478	0.148	0.017	0.261–0.874	0.2417	177
Leukemia	0.434	0.157	0.021	0.213–0.884	0.2417	90
Lymphoma	0.729	0.351	0.511	0.283–1.873	0.2679	65
Multiple Myloma	0.135	0.083	0.001	0.126–1.238	0.2803	22
**Neuro**	0.301	0.170	0.033	0.100–0.090	0.2818	25
**Sarcoma**	0.561	0.460	0.481	0.112–2.799	0.2624	22

A multivariate jackknife analysis method was employed using STATA Data Analysis and Statistical Software. N = 20 was the minimum number of samples set for analysis. ρ^2^ is known as pseudo R^2^. Note that the n column is not cumulative as "carcinoma" includes breast, prostate, and lung.

* Logistic regression sub-analysis was prostate was not possible; likely stemming from insufficient data.

**Table 3:** Coronary atherosclerosis, statistical results.

	Odds Ratio	Std Error	P	CI	ρ^2^	n
**Cancer**	0.772	0.157	0.204	0.518–1.151	0.1286	522
**Carcinoma**	0.596	0.145	0.033	0.370–0.960	0.1435	259
Breast	0.378	0.211	0.082	0.127–1.131	0.1946	23
Prostate	0.893	0.953	0.916	0.110–7.224	0.1699	22
Lung	2.165	1.639	0.307	0.491–9.544	0.1787	50
**Heme**	1.047	0.294	0.871	0.604–1.814	0.1586	177
Leukemia	0.763	0.259	0.425	0.392–1.483	0.2417	90
Lymphoma	3.429	1.98	0.033	1.106–10.632	0.1577	65
Multiple Myeloma	0.395	0.230	0.111	0.126–1.238	0.1886	22
**Neuro**	1.182	0.716	0.783	0.361–3.873	0.1869	25
**Sarcoma**	0.869	0.590	0.836	0.230–3.286	0.1733	22

A multivariate jackknife analysis method was employed using STATA Data Analysis and Statistical Software. N = 20 was the minimum number of samples set for analysis. ρ^2^ is known as pseudo R^2^. Note that the n column is not cumulative as "carcinoma" includes breast, prostate, and lung.

There appears to be a heterogeneous, correlative spread between atherosclerosis severity (with variance between aortic and coronary) and specific subgroups of cancers. Though this may be resultant of underlying/associative factors aside from the cancers themselves, we believe that this is not the case, and the differences are inherently tied to the cancer types themselves.

### SHRINE Results

Larger, multi-hospital cohort analysis revealed a significantly lower incidence of diagnosed atherosclerotic related conditions when compared to the control group (non-pathological fractures); the dataset is summarized in Table 4. This dataset was composed of a smaller subset of cancer types; these cancers were informed by our autopsy results and represented a spread between genders and the more clinically prevalent neoplasms. All prevalence rates for all cancer types when compared with the fracture group were highly significant (p-value <0.0001).

**Table 4:** Atherosclerotic prevalence in leukemia/lymphoma, lung, colorectal, breast, uterus/cervix, prostate cancer patients and control group: fractures; patients ≥ 50 years old.

	Subgroup	Prevalence (%) Within Total Athero	Prevalence (%) of Athero Within Total Subgroup
**Both**	**Leukemia/Lymphoma**	4.44	12.57
	**Lung**	5.35	17.63
	Colorectal*	5.54	18.17
**Female**	**Breast**	5.57	9.79
	**Uterus/Cervix**	1.41	11.47
**Male**	**Prostate**	5.86	18.40
**Out group**	**Fractures**	9.54	27.40

Prevalence (%) within total athero = (# of patients with specific cancer and athero) / (total # of patients with athero) * 100. Prevalence (%) of athero within total subgroup = (# of patient with specific cancer and athero) / (total # of patients with specific cancer) * 100.

* The colorectal data from BIDMC produced a fatal error in the union set search and was, therefore, excluded.

Patients in the fractures control group exhibited a diagnosed, non-coronary atherosclerotic disease rate of 27.40%. This value is nearly double all cancer groups that were tested; leukemia/lymphoma (12.57%), lung (17.63%), colorectal (18.17%), breast (9.79%), uterus/cervix (11.47%), and prostate (18.40%). Overall, there is a decreased incidence in all cancers as expected based on our autopsy results, however, we once again see a disparity between the reported values that likely stem from the differences between the cancers themselves.

## Discussion

It is apparent from our study that there exists an inverse relationship between cancer and atherosclerosis. Utilizing two sources of electronic medical records, we found that on a broad scale, patients of cancer are less likely to be clinically diagnosed for cardiovascular diseases while post-mortem analysis indicates that the severity of any present atherosclerotic disease is significantly diminished in such patients. We believe that the anti-inflammatory aspect of cancer’s pan-inflammatory response plays an important role towards atherosclerotic attenuation. Alternatively, the hypothesis of natural pro- versus anti-inflammatory physiology in an individual patient is supported by this data.

The seemingly forgotten notion that aspects of cancer may possess the ability to attenuate atherosclerosis provides a unique approach towards filling this gap in our knowledge between biology, pathology, and eventually novel therapies [13,14,15]. Likely stemming from the innate complexities and non-obvious associations between cancer and atherosclerosis, there is a distinct lack of knowledge regarding their influences on one another. Arguably, there are numerous factors clouding a clear path to causality. For instance, treatment and symptoms of cancer such as chemotherapy and cachexia have demonstrated the ability to reverse atherosclerosis [17–20]. Such effects, however, are not completely straightforward; reports have shown that certain chemotherapeutics can promote atherosclerosis, while the inflammatory nature of cachexia and wasting has been shown to aggravate lesion development [21,22]. Contrasting these thoughts, we believe that the underlying mechanism lies in possibly innate variations in human physiology or the direct effect of cancer biology—rather than in the treatments or symptomatic manifestations.

The correlation between coronary artery disease and atherosclerotic disease of the aorta was modest in our data set (0.5864), which reflects the known biological observation that patients may have a severe form of either with no relation to the other. Thus, patients with severe coronary artery disease leading to myocardial infarctions may have relatively clean aortic walls. To avoid loss of patients in our autopsy dataset, we imputed the aortic and coronary artery atherosclerosis scores from each other in a small number of cases. In doing so, the correlation in the data set remained the same (0.5864) and comparison of the pre- and post-imputation ORs for a given comparison showed little change except as noted above. This confirms that, despite the use of imputation, the data set we analyzed is essentially the same as the raw data set for the purposes of the associations we are comparing.

Several of the results with respect to our study are likely to be explained by presently known biology. Interestingly, our SHRINE results show that the fractures out-group had an atherosclerotic rate of 27.4%, which was incidentally very similar to reported values in our selected age group; this external validation adds confidence to our findings [23]. Our SHRINE dataset showed that prostate, lung, and colon cancer demonstrated essentially equivalent rates of atherosclerosis, 17.6–18.4%, which are less than fractures. We suspect that our anti-inflammatory hypothesis plays a role in these patients. We see that in our autopsy analysis, however, lung cancer has significantly elevated odds ratios, 1.801 in aortic and 2.165 in coronary atherosclerosis. We note the following may lead to the witnessed discrepancies and increased rate of atherosclerosis: a) lung cancer is strongly associated with smoking which aggravates atherosclerosis, b) instances of colon cancer are linked with a high fat diet which is also known to accentuate atherosclerosis [24,25].

Prostate cancer does not exhibit as pronounced an effect on atherosclerosis as other cancers. This may be due to that fact that prostate cancer is typically a disease of age and will thus have more overlap with similar temporally related diseases such as atherosclerosis. It should also be noted that our model could not produce an odds ratio association in our sub-analysis for aortic atherosclerosis due to low sample numbers. A larger sample set for this specific cancer is needed to address this issue. We confirmed that the inability to produce an odds ratio value was not due to co-linearity. Regression analysis between aortic atherosclerosis and prostate cancer values produced an R^2^ value of 0.01.

A reduced incidence of atherosclerotic disease and severity in breast and uterine/cervical cancer, seen in both autopsy and SHRINE analyses, comes as little surprise. Estrogen is known to be protective of atherosclerosis [26,27]. Estrogen likely explains the lower incidences of cardiovascular disease in women versus men. Cancers of the breast and uterus/cervix may result in an increased production of cellular derived estrogen resulting in an anti-atherosclerotic environment.

Results from the hematologic neoplasms proved to be the most unexpected and interesting. Autopsy analysis revealed odds ratios for aortic and coronary atherosclerosis of 0.434, 0.729, 0.135 and 0.763, 3.429, and 0.395 for leukemias, lymphomas, and multiple myeloma respectively. SHRINE data showed an incidence rate or 12.6%, which is less than prostate and sex-independent cancers and at a similar rate compared to female cancers.

There has yet to be a fully substantiated mechanism of action proposed for this phenomenon. We hypothesize that, due to cellular nature of these hematopoietic malignancies, an anti-inflammatory environment is promoted or perhaps existed in these patient’s baseline physiology, inherently leading to the reduction of any present atherosclerotic burden. What is unclear about this notion is whether released cytokines directly affect plaques or whether an indirect immune-modulation relationship is established eventually leading to an anti-atherosclerotic environment. For instance, it has been shown that regulatory T-cells are prevalent in acute myelogenous leukemia (AML) as a means for immune surveillance evasion [28]. It has also been demonstrated that regulatory T cells posses the ability to attenuate atherosclerosis [29, 30]. Thus, the increased regulatory T-cell recruitment may be one mechanism by which an anti-inflammatory physiology is obtained.

A rapidly expanding field, cancer immunology may provide illuminating insight into the herein reported phenomenon. A hallmark feature of cancer is its ability to evade the host immune system. This allows for unabated tumor expansion and increased potential for metastasis. It is reported that cancer is able to accomplish this feat through numerous mechanisms: downregulation of surface antigen presenting proteins, cytokine secretion, and reorganization of the local stromal environment. The predominant mechanism of action is thought to be resultant from cytokine production [31].

These cytokines are suggested to have broad reaching, immune-modulatory effects that have implications in atherogenesis. TGF-β and IL-10, for example, are two pleiotropic factors released by numerous cancers that have generalized anti-inflammatory properties. These two factors can act as T-cell and macrophage suppressors (cells able to eliminate cancer) while also promoting the development of regulatory T-cells (cells able to prevent tumor specific immune responses), as mentioned previously. There is concordant evidence that shifts in these particular immune populations are able to control atherosclerotic lesion development [28–34]. It should also be noted that cancer cells are well known to secrete pro-inflammatory cytokines such as TNF-α, IL-6, and IL-1β; all of which act to promote a local, pro-neoplastic environment (which may also influence atherosclerosis) [35]. It is still unknown whether these are the actual mechanism by which cancer is capable of modulating atherosclerosis, however, the pro/anti-inflammatory balance and immunological linkages between these two disease speaks to the feasibility of the notion.

Further studies have demonstrated that secreted factors from primary tumors and cancer cell lines are able to regulate T-cell associated inflammation. Several distinct cancer types interfere with NF-κB signaling, prompting anti-inflammatory activity. One study demonstrated that supernatant derived from AML cells inhibit T-cell nuclear translocation of NF-κB with associated reductions in IL-2 and IFN-y production [36]. A similar reduction in NF-κB translocation was seen in renal cell carcinomas [37]. NF-κB is a known regulator of atherosclerotic cytokines such as IL-6, TNF-α, and IL-1β. Thus, NF-κB modification may be a potential mechanism by which lesion attenuation is achieved via a diminishment in pro-atherosclerotic cytokines. It would be of interested to investigate whether similar effects were achieved in other prominent cells found in atherosclerotic lesions such as monocytes and macrophages.

There were several issues with respect to our study that we were unable to fully resolve. One such factor was the temporal component of patients’ cancer burdens; this is clearly an issue experienced by the clinical cancer field as a whole. Our hypothesis revolves around the notion that cancer arises from and promotes a global anti-inflammatory physiology. We postulate that those with longer courses of cancer burden are likely to have an extensive molecular, anti-inflammatory signature with associated reduced atherosclerotic burden compared to those with shorter cancer courses. Presently, determining the exact timeframe of cancer in humans is not possible on a clinical scale. Any further experimentation on this phenomenon will likely be most fruitful *in vivo* where cancer induction and tracking can be rigorously controlled and monitored.

Another clinical aspect we were unable to fully resolve was chemotherapy. We did not exclude/include patients based on surgical cure, presence/absence of chemotherapy, or palliative care only status. Thus, our sampling represents the full spectrum of whatever effect chemotherapy may have on atherosclerosis. With the numerous forms of chemotherapy compounded with various treatment durations, inclusion and analysis proved to be intangible. Given that such treatments are historically aimed towards rapidly dividing cells, unlike resident atherosclerotic foam cells, it does not seem likely that diminished atherosclerosis rates in this study in the timescale of treatment for cancer is due to chemotherapeutics. Furthermore, reversibility in the dynamics of atherosclerotic disease occurs in early lesions and at the coronary level but has not been demonstrated in large complex plaque lesions [38–40].

The discrepancies between aortic and coronary atherosclerosis also presented an interesting result. The locational differences clearly result in drastic pathology spanning myocardial infarction to stroke. Although the effect of soluble cancer factors may systemic, such observed differences may be due to the underlying variations in hemodynamics. Because the autopsy data was incomplete and, thus, imputation was used for statistical purposes, we cannot fully evaluate the specific relationships between coronary or aortic atherosclerosis independently in our data set.

The herein results represents one of the only recent reports that addresses the inverse relationship between cancer and atherosclerosis. More exhaustive studies are clearly necessary in order to truly understand the mechanisms behind this phenomenon. We believe that this novel paradigm will uncover novel biology towards a greater understanding, and eventually treatment, of atherosclerotic disease.

## Supporting Information

S1 Table The primary outputs are shown for all cancers in the multivariate models including aortic or coronary are shown for non-imputed (raw) data by each diagnosis type with a corresponding optimized model (where non-selected co-variates are removed). (XLSX)

S2 Table The primary outputs are shown for all cancers in the multivariate models including aortic or coronary are shown for imputed data by each diagnosis type with a corresponding optimized model (where non-selected co-variates are removed). (XLSX)

S3 Table The summarized odds ratios are shown of all models (output = cancer type) for non-imputed and imputed data for aortic and coronary disease. (XLSX)

## References

[1] World Health Organization, *Cardiovascular disease (CVDs)*, http://www.who.int/mediacentre/factsheets/fs317/en/index.html.

[2] Mayo Clinic, *Heart Disease*: *Treatments and drugs*, http://www.mayoclinic.com/health/heart-disease/DS01120/DSECTION=treatments-and-drugs.

[3] Scandinavian Simvastatin Survival Study Group, *Randomised trial of cholesterol lowering in 4444 patients with coronary heart disease*: *the Scandinavian Simvastatin Survival Study (4S)* , The Lancet, 344:1383–89 (1994). 7968073

[4] Cholesterol Treatment Trialists’ (CTT) Collaborators, *Efficacy and safety of cholesterol-lowering treatment*: *prospective meta-analysis of data from 90 056 participants in 14 randomised trials of statins* . The Lancet, 366:1267–78 (2005). 16214597 10.1016/S0140-6736(05)67394-1

[5] Steinberg D, *Thematic review series*: *The Pathogenesis of Atherosclerosis*. *An interpretive history of the cholesterol controversy*: *part I* , The Journal of Lipid Research, 45:1583–1593 (2004). 15102877 10.1194/jlr.R400003-JLR200

[6] Forrester JS, Bairey-Merz CN, Kaul S, The aggressive low density lipoprotein lowering controversy, Journal of the American College of Cardiology, 36(4):1419–1425 (2000). 11028504 10.1016/s0735-1097(00)00829-9

[7] Hayward RA, Hofer TP, Vijan S, Narrative review: lack of evidence for recommended low-density lipoprotein treatment targets: a solvable problem, Ann Intern Med, 145(7):520–30 (2006). 17015870 10.7326/0003-4819-145-7-200610030-00010

[8] Jain MK, Ridker PM, Anti-Inflammatory Effects of Statins: Clinical Evidence and Basic Mechanisms, Nature Reviews Drug Discovery, 4:977–987 (2005). 16341063 10.1038/nrd1901

[9] Yaakov Henkin, Re-Evaluating Therapeutic Target Goals for Statin-Treated Patients, Journal of the American College of Cardiology, 52(8) (2008). 10.1016/j.jacc.2008.05.002 18702966

[10] Weitz-Schmidt G, Statins as anti-inflammatory agents, Trends in ParmacologicalSceinces, 23(10):482–487 (2002). 12368073 10.1016/s0165-6147(02)02077-1

[11] Sparrow CP, Burton CA, Hernandez M, Mundt S, Hassing H, Patel S, et al . ., Simvastatin Has Anti-Inflammatory and Antiatherosclerotic Activities Independent of Plasma Cholesterol Lowering, Arteriosclerosis, Thrombosis, and Vascular Biology, 23:115–121 (2001). 10.1161/01.atv.21.1.115 11145942

[12] Ridker PM, Rifai N, Rose L, Buring JE, Cook NR, Comparison of C-Reactive Protein and Low-Density Lipoprotein Cholesterol Levels in Prediction of First Cardiovascular Events, The New England Journal of Medicine, 347:1557–1565 (2002). 12432042 10.1056/NEJMoa021993

[13] Elkeles A, Cancer and Atherosclerosis, British Journal of Cancer, 10(2):247–250 (1956). 13364114 10.1038/bjc.1956.28 PMC2073800

[14] Elkeles A, Calcified Atherosclerosis and Cancer, British Journal of Cancer, 13(3):403–407 (1959). 13819947 10.1038/bjc.1959.46 PMC2074099

[15] Winklestein R, Lilienfeld R Jr., Pickren JW, Lilienfeld AM, The Relationship Between Aortic Atherosclerosis and Cancer, British Journal of Cancer, 13(4):606–613. 13845253 10.1038/bjc.1959.65 PMC2074177

[16] McFadden D, Quantitative methods for analyzing travel behavior of individuals: Some recent developments, Cowles Foundation discussion paper no. 474, Yale University, New Haven, CT (1977).

[17] Spain DM, Greenblatt IJ, Snapper I, Cohn T, The degree of coronary and aortic atherosclerosis in necropsied cases of multiple myeloma, The American Journal of Medical Sciences, 231(2):165–7 (1956). 13282898

[18] Numano F, Chemotherapy of atherosclerosis, Japan Circulation Journal, 44(1):55–68 (1980). 6245288 10.1253/jcj.44.55

[19] Maniscalco BS, Taylor KA, Calcification in coronary artery disease can be reversed by EDTA-tetracycline long-term chemotherapy, Pathophysiology, 11(2):95–101 (2004). 15364120 10.1016/j.pathophys.2004.06.001

[20] Wilens SL, The resorption of arterial atheromatous deposits in wasting disease, American Journal of Pathology, 23(5):793–804 (1947). 19970960 PMC1934310

[21] Skeijima T, Tanabe A, Maruoka R, Fijishiro N, Yu S, Fujiwara S, et al, Impact of platinum-based chemotherapy on the progression of atherosclerosis, Climacteric, 14(1):31–40 (2011). 10.3109/13697137.2010.522278 21067421

[22] Zyga S, Christopoulou G, Malliarou M, Malnutrition-Inflammation-Atherosclerosis Syndrome in Patients With End-Stage Renal Disease, Journal of Renal Care, 37(1):12–15 (2011). 10.1111/j.1755-6686.2011.00201.x 21288312

[23] Jaffer FA, O’Donnell CJ, Larson MG, Chan SK, Kissinger KV, Kupka MJ, et al, Age and Sex Distribution of Subclinical Aortic Atherosclerosis: A Magnetic Resonance Imaging Examination of the Framingham Heart Study, Arteriosclerosis, Thrombosis, and Vascular Biology, 22:849–854 (2002). 12006401 10.1161/01.atv.0000012662.29622.00

[24] Howard G, Wagenknecht LE, Burke GL, Diez-Roux A, Evans GW, McGovern P, et al GS,Cigarette Smoking and Progression of Atherosclerosis. The Journal of the American Medical Association, 308(24):2577–2583 (1998). 10.1001/jama.279.2.119 9440661

[25] Slattery ML, Boucher KM, Caan BJ, Potter JD, Ma KN,Eating Patterns and Risk of Colon Cancer. American Journal of Epidemiology, 148(1):4–16 (1998). 9663397 10.1093/aje/148.1.4-a

[26] Marsh MM, Walker VR, Curtiss LK, Banka CL,Protection against atherosclerosis by estrogen is independent of plasma cholesterol levels in LDL receptor-deficient mice. Journal of Lipid Research, 40:893–900 (1999). 10224158

[27] Nathan L, Chaudhuri G, *Estrogens and Atherosclerosis*, Annual Reviews Pharmacology and Toxicology, 37:477–515 (1997). 9131262 10.1146/annurev.pharmtox.37.1.477

[28] Ustun G, Miller JS, Munn DH, Weisdorf DJ, Blazar BR, Regulatory T cells in acute myelogenous leukemia: is it time for immunomodulation?,Blood, 118(19):5084–5095 (2011). 10.1182/blood-2011-07-365817 21881045 PMC3217399

[29] Mallat Z, Ait-Oufella H, Tedgui A, Regulatory T-Cell Immunity in Atherosclerosis, Trends in Cardiovascular Medicine, 17(4):113–118 (2007). 17482092 10.1016/j.tcm.2007.03.001

[30] Ait-Oufella H, Salomon BL, Potteaux S, Robertson AKL, Gourdy P, Zoll J, et al, Natural regulatory T cells control the development of atherosclerosis in mice, Nature Medicine, 12,178–180 (2006). 16462800 10.1038/nm1343

[31] Zindl CL, Chaplin DD, Tumor Immune Evasion, Science, 329(5979):697–698 (2010). 10.1126/science.1190310 20448171 PMC4123868

[32] Topfer K, Kempe S, Muller N, Schmitz M, Bachmann M, Cartellieri M, et al, Tumor Evasion from T Cell Surveillance, Journal of Biomedicine and Biotechnology, (2011). 10.1155/2011/918471 PMC3228689 22190859

[33] Ammirati E, Cianflone D, Vecchio V, Banfi M, Vermi A, De Metrio M, Grigore L, et al, Effector Memory T cells Are Associated With Atherosclerosis in Humans and Animal Models, Journal of the American Heart Association, 1:27–41 (2012). 10.1161/JAHA.111.000125 23130116 PMC3487313

[34] Mallat Z, Besnard S, Duriez M, Deleuze V, Emmanuel F, Bureau MF, Soubrier F, et al, Protective role of interleukin-10 in atherosclerosis, Circulation Research, 15;85(8):e17–24 (1999). 10521249 10.1161/01.res.85.8.e17

[35] Coussens LM, Werb Z, Inflammation and cancer, Nature, 420:860–867 (2002). 12490959 10.1038/nature01322 PMC2803035

[36] Buggins GS, Milojkovic D, Arno MJ, Lea NC, Mufti GJ, Thomas NSB, et al Microenvironment Produced by Acute Myeloid Leukemia Cells Prevents T Cell Activation and Proliferation by Inhibition of Nf-kB, c-Myc, and pRb Pathways, The Journal of Immunology, 167: 6021–6030 (2001). 11698483 10.4049/jimmunol.167.10.6021

[37] Kim HJ, Park JK, Kim YG, Suppression of NF-kB Activation in Normal T Cells by Supernatant Fluid from Human Renal Cell Carcinomas, Journal of Korean Medical Science, 14:299–303 (1998). 10.3346/jkms.1999.14.3.299 PMC3054373 10402173

[38] Poli A, Corsini A. Reversible and non-reversible cardiovascular risk in patients treated with lipid-lowering therapy: analysis of SEAS and JUPITER trials. Eur J Intern Med. 2010 10;21(5):372–3. 10.1016/j.ejim.2010.04.013 Epub 2010 Jul 3. 20816586

[39] Oka K, Chan L. Inhibition and regression of atherosclerotic lesions. Acta Biochim Pol. 2005;52(2):311–9. Epub 2005 Jun 3. 15940346 PMC1360606

[40] Sacks FM, Gibson CM, Rosner B, Pasternak RC, Stone PH. The influence of pretreatment low density lipoprotein cholesterol concentrations on the effect of hypocholesterolemic therapy on coronary atherosclerosis in angiographic trials. Harvard Atherosclerosis Reversibility Project Research Group. Am J Cardiol. 1995 9 28;76(9):78C–85C. 7572692 10.1016/s0002-9149(99)80475-5

